# Influence of Architecture of β-Tricalcium Phosphate Scaffolds on Biological Performance in Repairing Segmental Bone Defects

**DOI:** 10.1371/journal.pone.0049955

**Published:** 2012-11-21

**Authors:** Ya-Fei Feng, Lin Wang, Xiang Li, Zhen-Sheng Ma, Yang Zhang, Zhi-Yong Zhang, Wei Lei

**Affiliations:** 1 Department of Orthopedics, Xijing Hospital, The Fourth Military Medical University, Xi’an, China; 2 School of Mechanical Engineering, Shanghai Jiao Tong University, State Key Laboratory of Mechanical System and Vibration, Shanghai, China; 3 Department of Plastic and Reconstructive Surgery, Shanghai 9th People’s Hospital, Shanghai Key Laboratory of Tissue Engineering, School of Medicine, Shanghai Jiao Tong University, Shanghai, China; 4 National Tissue Engineering Center of China, Shanghai, China; National University of Ireland, Ireland

## Abstract

**Background:**

Although three-dimensional (3D) β-tricalcium phosphate (β-TCP) scaffolds serve as promising bone graft substitutes for the segmental bone defect treatment, no consensus has been achieved regarding their optimal 3D architecture.

**Methods:**

In this study, we has systematically compared four types of β-TCP bone graft substitutes with different 3D architectures, including two types of porous scaffolds, one type of tubular scaffolds and one type of solid scaffolds, for their efficacy in treating segmental bone defect in a rabbit model.

**Results:**

Our study has demonstrated that when compared to the traditional porous and solid scaffolds, tubular scaffolds promoted significantly higher amount of new bone formation in the defect regions as shown by X-ray, micro CT examinations and histological analysis, restored much greater mechanical properties of the damaged bone evidenced by the biomechanical testing, and eventually achieved the complete union of segmental defect. Moreover, the implantation of tubular scaffolds enhanced the neo-vascularization at the defect region with higher bone metabolic activities than others, as indicated by the bone scintigraphy assay.

**Conclusions:**

This study has further the current knowledge regarding the profound influence of overall 3D architecture of β-TCP scaffolds on their in vivo defect healing performance and illuminated the promising potential use of tubular scaffolds as effective bone graft substitute in treating large segmental bone defects.

## Introduction

Treatment of large segmental bone defects caused by severe trauma, non-union fractures or tumor resection remains as a major clinical challenge. Nowadays, the usual strategy for large bone defect treatment involves the use of autologous or allogeneic bone grafts, with more than 500,000 bone grafting procedures conducted annually in the United States alone, making bone tissue the second largest transplanted tissue in the world [1]. The autologous bone grafts, which are considered to be the gold standard [2], require two seperate surgical operations associated with various complications such as wound dehiscence, vessel injuries, hematoma and infections [3], [4], [5]. The use of allografts can avoid the secondary surgery, nevertherless, is harrased by the concerns of disease transmission and decreased donor tissue sources [6]. The development of three-dimensional (3D) synthetic scaffold as bone graft substitutes has become the promising attempt to overcome these limitations and eventually addressed the ever-increasing clinical need for large bone defect treatment [4], [7], [8]. A suitable 3D scaffold for bone defect treatment should be able to fullfil the following criteria: (1) provide the initial mechanical support to protect the defect area from the collapse of surrounding tissue; (2) be able to prevent the invasion of fibrous tissue; (3) possess favorable osteoconductivity to promote bone tissue ingrowth; (4) allow sufficient vascularization within the constructs to promote the new bone regeneration [9].

In order to fabricate a favorable 3D scaffold as bone graft substitute to promote bone defect healing, various types of materials with different chemical properties were carefully investigated, including metal, polymeric material, ceramic material and so on. Bioactive ceramic materials such as β-tricalcium phosphate (β-TCP) have been demonstrated as attractive material candidates for 3D scaffold fabrication, because of their chemical similarity to the inorganic phase of natural bone, favorable biocompatibility, osteoconductivity and bioresorbable properties [10], [11], [12], [13]. Besides the chemical properties of material for fabrication, the physical characteristics, especially the 3D architecture of the constructs have been proven to be another determinant for developing a suitable bone graft substitute, which influenced in vivo performance of 3D constructs profoundly [14]. However, there has been still no consensus regarding the optimal architecture of 3D scaffolds as bone substitutes for bone regeneration and vascularization. Porous architecture has been widely used for the fabrication of 3D scaffold, because it is similar to the nature structure of bone tissue, allowing certain degree of bone tissue infiltration and vascularization without compromising the mechanical property [15], [16]. The detailed architecture characteristics of porous scaffolds such as porosity and pore size have been intensively investigated for their influence on the in vivo bone tissue ingrowth and vascularization [17], [18]. But the use of porous scaffolds as bone substitutes for large segmental bone defect treatment is usually hindered by the limited bone ingrowth, especially in the center region of grafts [19]. On the other hand, tubular design has been proposed in several studies as well, in order to mimic the tubular structure of long bone, with potential efficacy to facilitate bone tissue infiltration and vascularization [20], [21]. However, how the tubular scaffolds compared to porous ones as bone graft substitutes in terms of their osteoconductivity (bone tissue ingrowth) and vascularization during the defect treatment still remains elusive. Therefore, in current study, we conducted a head-to-head comparison of β-TCP scaffolds with different porous architecture as well as tubular architecture for their in vivo performance. Firstly, we precisely designed and fabricated four types of β-TCP constructs with different architectures, then implanted them into a rabbit radius defect model for 12 weeks and evaluated the influence of their architecture on the infiltration of fibrous tissue, bone tissue ingrowth, vascularization and in vivo mechanical properties.

## Materials and Methods

### Fabrication and Characterization of β-TCP Scaffolds

Commercial β-TCP powder was obtained from Edward Keller (Shanghai, China). The β-TCP scaffolds were manufactured as described previously [11]. In brief, large β-TCP cylinders were designed with computer-aided design (CAD) software. The epoxy molds were made by stereolithography (SL), one of the solid freeform fabrication (SFF) techniques. Prepared β-TCP suspension was cast into the molds and sintered at 1100°C, so that the epoxy mold with interconnected beams can be removed by pyrolysis to create the channel space in the scaffolds. Four types of cylindrical β-TCP scaffolds were fabricated with the diameter of 4 mm and the height of 16 mm. The morphology and 3D structure of the scaffolds were characterized by micro-computerized tomography (micro-CT) (eXplore Locus SP, GE Healthcare, Canada). The β-TCP scaffolds were individually double packed and steam sterilized at 121°C for 20 min before implantation.

### Animal Study

#### Animals ethics and experiment design

**Figure 1:**
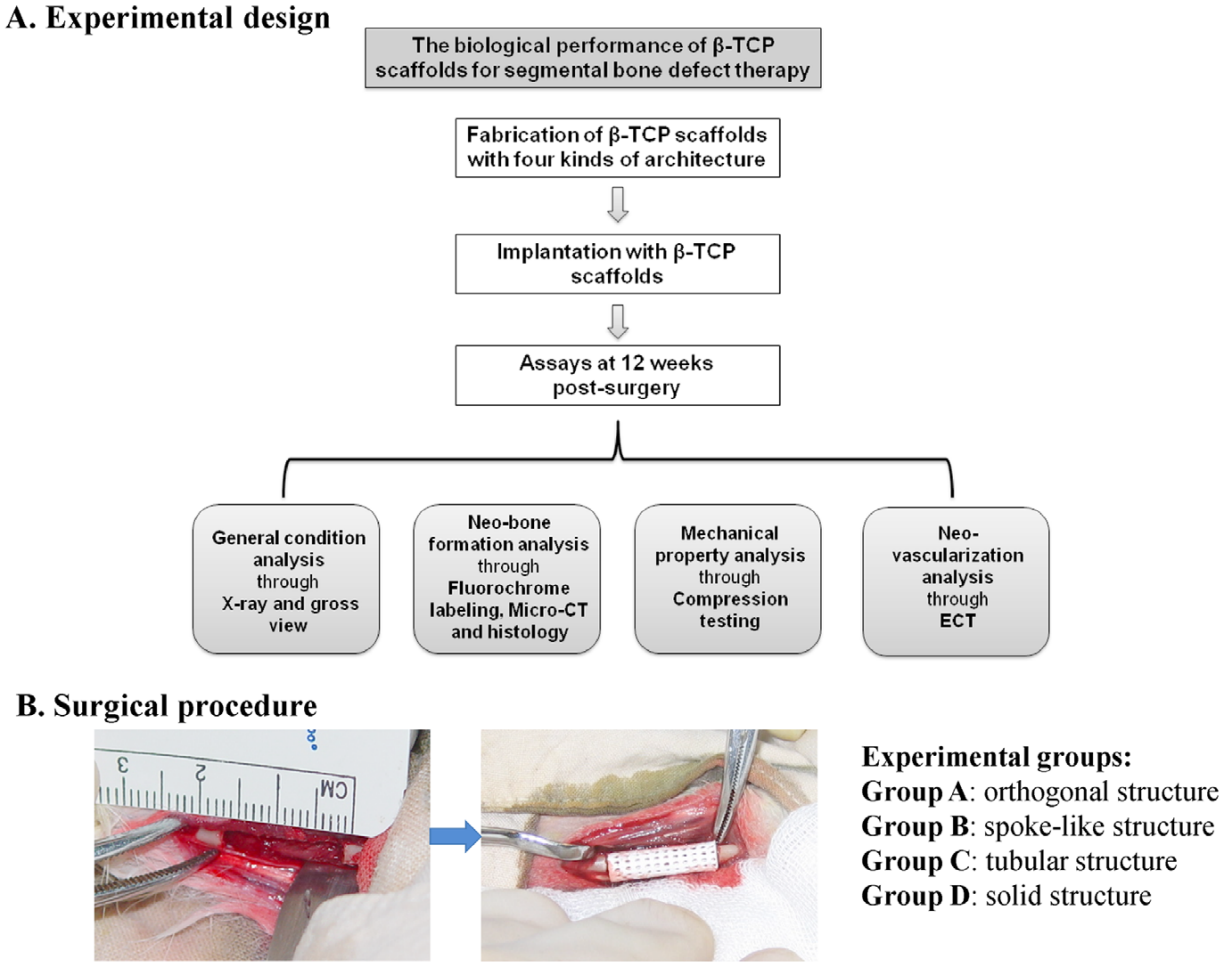
Experimental design and surgical procedure. (**A**) The experimental design of the whole study. (**B**) A 16 mm-long segmental defect was created on the radius of each rabbit and implanted with four types of β-TCP scaffolds with different architectures.

All animal experiments were performed in strict accordance with protocols approved by the Institutional Animal Care Committee of Xijing Hospital. Forty New Zealand rabbits (Male, 6 months age, 3.5–3.75 kg) were randomly divided into six experimental groups: (Group A-D) β-TCP scaffold group (n = 8 in each group), in which defects were implanted with four types of β-TCP scaffolds respectively according to a randomized complete block design; (Group E) autologous bone control group (n = 4), in which defects were implanted with autologous radius; and (Group F) control untreated group (n = 4), in which defects were left untreated to validate that the defects created here are critical sized defect. In order to analyze the biological performance of β-TCP scaffolds for large bone defect treatment, X-ray, gross view, fluorochrome marker, micro-computer tomography (micro-CT), biomechanical testing, histology and emission computed tomography (ECT) analysis were carried out in the present study (Fig. 1).

#### Surgical procedure

General anesthesia was performed on rabbits before the surgery using an intramuscular injection of a mixture of Ketamine and Xylazine. Limbs were shaved, skin was disinfected with iodine solution and a longitudinal incision was made to expose the radius, and a 16-mm segmental bone defect was created with a miniature oscillating saw [22]. The cylindrical β-TCP scaffolds or autologous bone grafts were then press-fitted into the radius defect (Fig. 1B). The subcutaneous tissue and skin were closed in layers using degradable sutures. The surgical site was finally covered with an adhesive bandage. Each rabbit was administered 400,000 U of penicillin intraoperatively and on the first postoperative day to prevent infection. At the predetermined time point, the animals were anaesthetized and sacrificed by intra-cardiac overdose of sodium pentobarbital. The middle radius were immediately dissected and prepared for testing.

#### Radiographic imaging and gross view

At 12 weeks post-surgery, under the anesthesia, the defect region of rabbits was subject to the radiographic imaging (distance: 1 mm; X-ray source: 46 Kv, 50 mA; exposure time: 0.14 s) to assess the healing of bone defect. The status of bone repair and growth of callus were studied in samples taken out through the original incision after the animals were sacrificed.

##### Fluorochrome labeling

Sequential fluorochrome markers were administered 2 weeks and 3 days before the animals were sacrificed. Tetracycline (30 mg/kg, Sigma, USA) was administered using intramuscular injection. After the animals were sacrificed at 12 weeks post-surgery, the implants were retrieved for fluorescence analysis.

##### Micro-CT analysis

Specimens containing the scaffold construct and some surrounding tissue were harvested (n = 8 in Group A-D) and fixed in 10% neutral buffered formalin, placed in the sample holder and scanned under the micro CT (eXplore Locus SP, GE Healthcare, Canada). About 1600 projections of 1024^2^ pixels were acquired for each tomogram. The X-ray source voltage was set at 80 kV and beam current at 200 mA using filtered Bremsstrahlung radiation. The scanning angular rotation was 180°, and the angular increment was 0.40°. To minimize beam hardening artifacts a 1 mm aluminum X-ray beam filter was used to attenuate soft X-rays at the source. The projections were reconstructed using a modified parallel Feldkamp algorithm, and segmented into binary images (8-bit BMP images). For determination of the 3D micro-architectural properties within the bone regeneration area, specimens were evaluated using 3D analysis software (Microview, GE Healthcare, Canada). The percentage of bone volume out of total volume (BV/TV) and degradation rate were calculated using the threshold of 2000 for bone tissue and 3000 for scaffold.

##### Biomechanical testing

Immediately after the animals were sacrificed, the mechanical properties of the implants (n = 4 in Group A–D) were evaluated with the use of a destructive compression test. Cubes of 8mm×4mm×4mm from each specimen were sawed and tested under wet conditions at room temperature. The compressive load was applied at cross-head speed of 1 mm/min using the material testing machine (AGS-10kN, Shimadzu Co., Kyoto, Japan) until fracture occurred. From the load-displacement curve, the compressive strength was calculated from compressive load and geometric area of the specimens. The compression test of the β-TCP scaffold was also done before implanted.

##### Histology analysis

Specimens from bone defect sites (n = 4 in Group A–D) were fixed in 10% formalin solution for 7 days. All implants were dehydrated in a graded ethanol series (70–100%) and transferred into a methylmethacrylate (MMA) solution that polymerized at 37°C within 1 week. Three slices of histology section (about 50 µm in thickness) were conducted longitudinally alone the long axis of tibia at the central region using the modified interlocked diamond saw (Leica Microtome, Wetzlar, Germany). Histological sections remained unstained first and were subjected to the fluorescence analysis using epifluorescence microscopy. Then they were stained with 1.2% trinitrophenol and 1% acid fuchsin (Van Gieson staining) for light microscopy analysis. The qualitative analysis of bone formation and fluorochrome markers were performed using a light/fluorescence microscope (Leica LA Microsystems, Bensheim, Germany). Prior to histomorphometry analysis, bone and material were pseudocoloured respectively using Adobe Photoshop 6.0 and then measured using an image analysis system (Image-Pro Plus software, Media Cybernetics, Silver Spring, USA). Bone formation was quantified from the pixels that represented bone tissue, while the total area was defined as the implanted bone site (4 mm×16 mm). The rate of new bone formation was presented as the percentage of bone area in total implant area [(bone area/total area)×100%]. In addition, the bone mineralization apposition rate (MAR, vertical spacing between two fluorochrome markers/injection interval) was analyzed from the images of fluorochrome labeling.

##### ECT analysis

ECT was performed at 2, 4, 8 and 12 weeks after surgery. ^99m^Tc-methylene- diphosphonate (MDP) was injected through the ear vein at a dose of 5 MBq/kg. ECT images were acquired (140 Kev maximum) 4 h after injection, and delayed images were obtained (512×512 acquisition matrix). During the dynamic imaging, manually drawn rectangular regions of interest (ROIs) (1cm×1cm, 40 pixels) were established on the implanted bone sites. The uptake ratio of ^99m^Tc-MDP (T/NT) between the implant site and the contralateral radius was determined for quantitative analysis.

##### Statistical analysis

The results were expressed as the means ± SD. Statistical analysis was performed using one way ANOVA followed by a SNK-q test for multiple comparisons. Values of *p*<0.05 were considered to be statistically significant.

## Results

### Characterization of β-TCP Scaffolds

**Figure 2:**
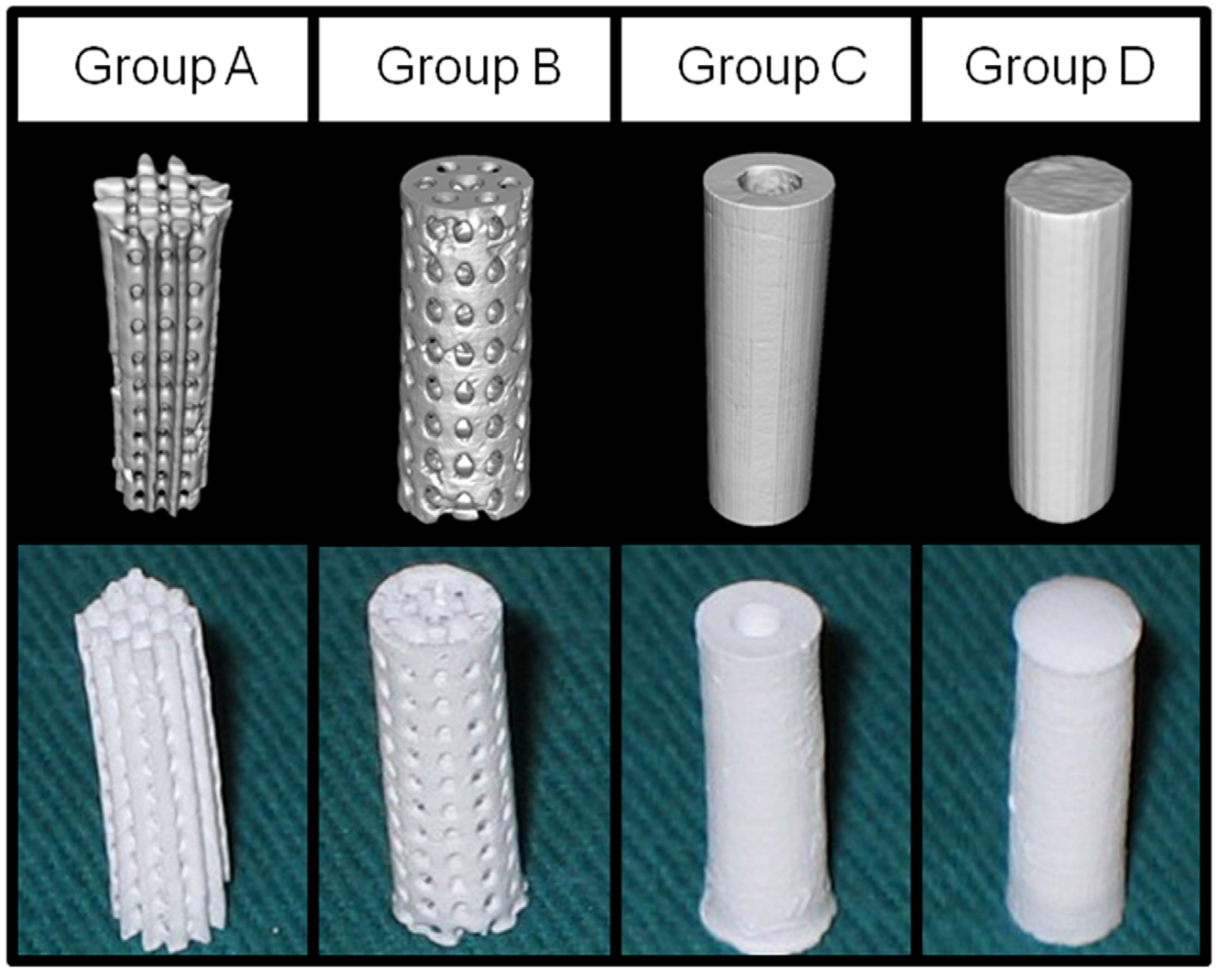
Characterization of β-TCP scaffolds. Micro-CT 3D image (upper row) and gross view (lower row) showed the general constructions of the four types of scaffolds. Two porous scaffolds (Group A and B) with porosity of around 40% and pore size of 500 µm, the tubular scaffolds (Group C) with inner diameter of 2 mm and the solid scaffolds (Group D) were manufactured and used in this study.

Four types of cylindrical β-TCP scaffolds with different architectures were manufactured and characterized by micro-CT respectively (Fig. 2). Two types of porous scaffolds were fabricated to mimic the structures of human trabecular bone: the orthogonal structure scaffold (Group A, Fig. 2) had the porosity of around 42% and the pore size of about 500 µm; the spoke-like structure scaffold (Group B, Fig. 2) had the porosity of around 40% and pore size of about 500 µm. The third type of scaffold was tubular shaped (Group C, Fig. 2) with inner diameter of 2 mm, mimicking the tubular structure of long bone. The forth type of scaffold was solid cylindrical (Group D, Fig. 2) as a control.

### X-rays Examinations and Gross Observation

**Figure 3:**
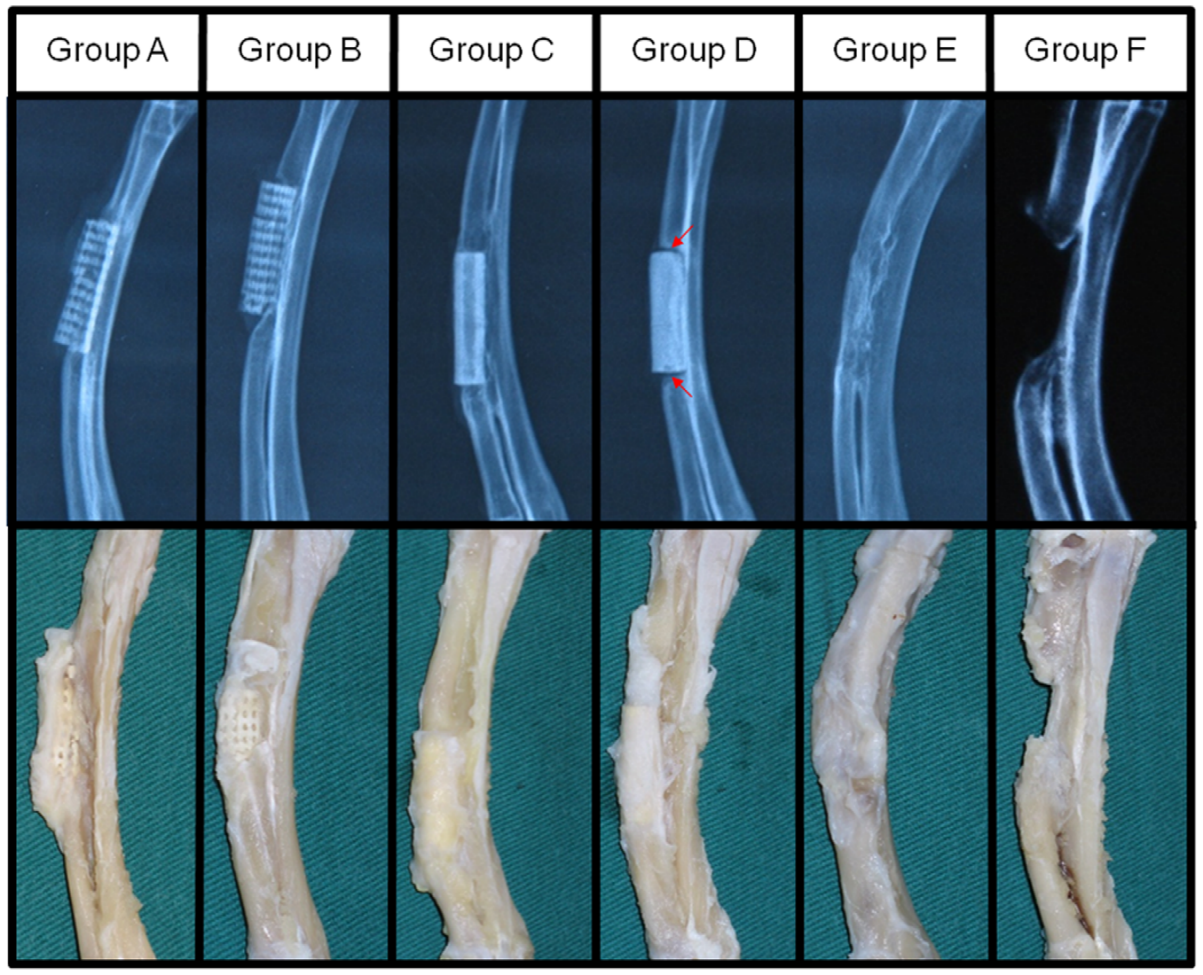
X-ray and gross examinations of the radius with different treatment at 12 weeks post-surgery. X-ray examination (upper row) showed evident ingrowth of new bone tissue into the porous (Group A and B) and tubular (Group C) scaffolds. Tubular scaffolds showed better graft/bone integration than porous ones, which was comparable to the implantation of autologous bone graft (Group E). The solid scaffolds (Group D) showed the poorest osteointegration as evidenced by clear boundary line (red arrow) between the scaffold and bone tissue. Non-union healing of the bone defect in Group F (no treatment) validated that the segmental defect used in this study was the critical sized bone defect. Gross view of the radius (lower row) showed treatment with porous or tubular scaffolds and autologous bone graft exerted better bony union than solid scaffolds.

[TIGHTER All rabbits exhibited normal diets and movements after surgery, without any surgical complications and sign of infection. X-ray images were taken to evaluate the position of the scaffolds and the development of bone regeneration within the defects (Fig. 3, the upper row). At 12 weeks postoperatively, the newly formed bone tissue infiltrated and integrated well with the scaffold constructs in Group A–C, which was similar to the complete integration found between implanted autologous bone graft and surrounding bone tissue in Group E. Whereas, Group D showed limited osteointegration between the solid scaffold and the surrounding bone tissue, as evidenced by the clear boundary line observed (Fig. 3, red arrow) between the implants and bone tissue. Group F led to the non-union healing of the bone defect, validating that the segmental bone defect created in this study was the critical-sized bone defect.

In agreement with the radiographic analysis, the gross images of the radius repaired with the implantation of porous and tubular β-TCP constructs (Group A–C) and autologous bone graft (Group E) showed well integration of implants with the surrounding tissue (Fig. 3, the lower row). In Group D, the radius, which was implanted with solid β-TCP constructs, achieved partial bony union and limited bone defects healing. In Group F, the defects were filled with fibrous-like tissue and the medullary cavities were blocked.

### Fluorochrome Labeling

**Figure 4:**
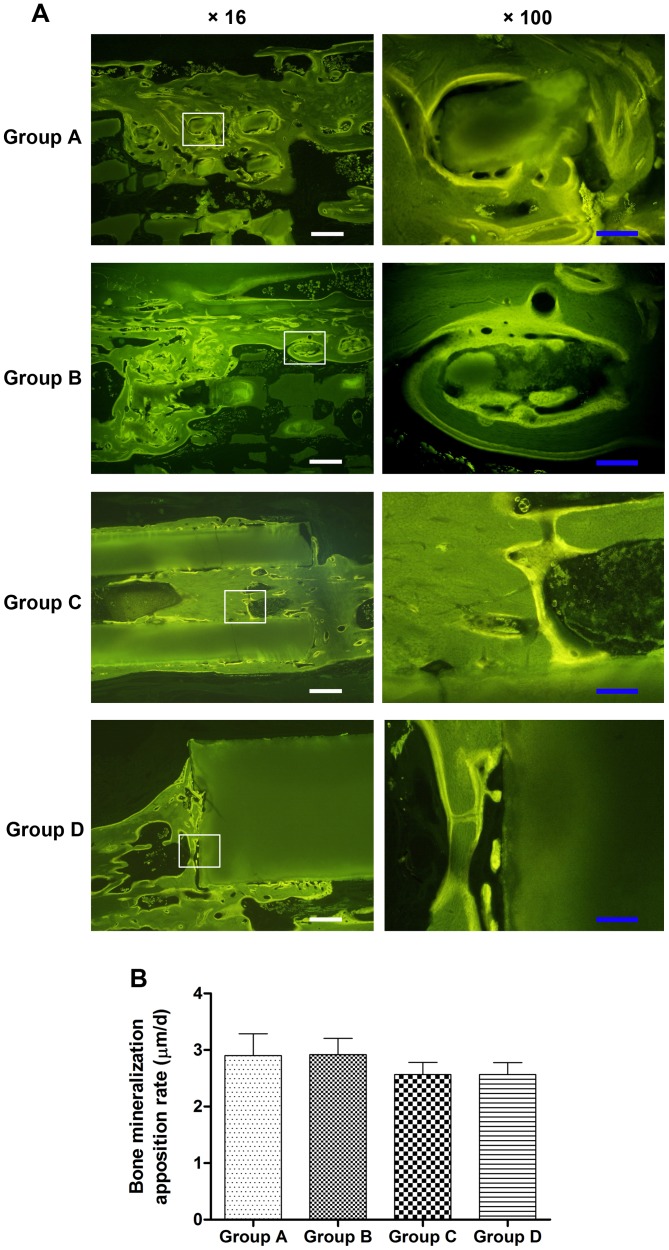
Fluorochrome labeling of bone regeneration at 12 weeks post-surgery. (**A**) The fluorescent labeling images indicated that more new bone growth into the porous (Group A–B) and tubular scaffolds (Group C), while very limited bone tissue formed around the solid scaffolds (Group D). (**B**) Quantitative analysis showed there were no significant differences in the bone mineralization apposition rate (*p*>0.05), indicating similar bone growth rate in these four kinds of scaffolds. Scale bar: 50 µm (white), 10 µm (blue).

Fluorescent labeling was detected in the regenerated bone in all specimens. Bone remodeling was validated by interrupted bone labeling and new bone deposition. The bone labeling was detected in the pores of both porous scaffolds (Group A–B) and tubular scaffolds (Group C), while only limited amount of new bone formed around the solid scaffolds (Group D) (Fig. 4A). It was noted that most of the newly formed bone was observed on the peripheral area rather than the center area of porous scaffolds. The quantitative analysis of fluorochrome markers (Fig. 4B) showed that the bone MAR of porous scaffolds (Group A and B) was slightly higher than that of tubular and solid scaffolds (Group C and D) but without statistically significant difference (*p*>0.05), indicating a similar growth rate of bone tissue in these four kinds of scaffolds.

### Micro-CT Evaluation

**Figure 5:**
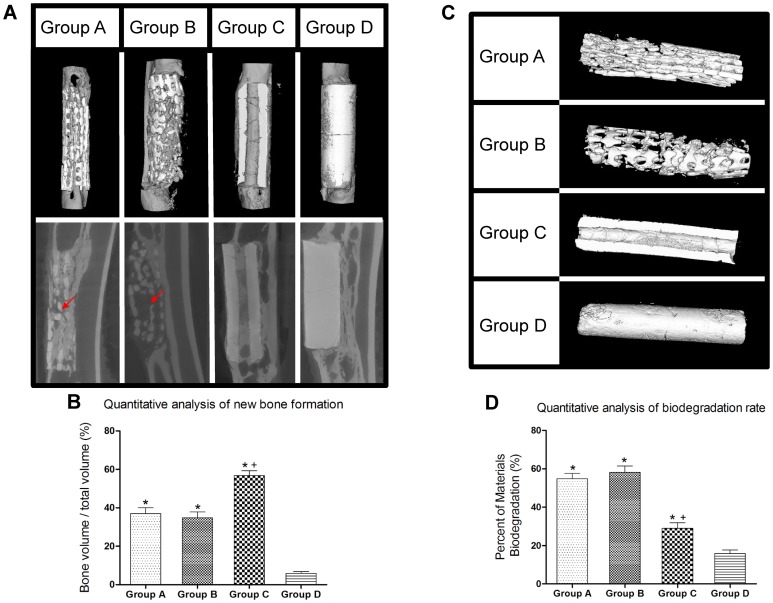
Micro-CT analysis of new bone formation and biodegradation rate of scaffolds at 12 weeks post-surgery. (**A**) Micro-CT images showed more bone ingrowth within the tubular scaffolds (Group C) than porous ones (Group A and B), especially in the center of grafts (red arrow). Little new bone generated in solid scaffolds (Group D). (**B**) The ratio of bone volume/total volume (%BV/TV) in Group C was higher than that of Group A, B and D. (**C**) Micro CT images indicated that porous scaffolds experienced faster degradation rate with ruptured overall structure, while tubular scaffolds maintained intact for its overall structure. (**D**) Quantitative analysis confirmed lower biodegradation rate in tubular scaffolds (Group C) than porous scaffolds (Group A and B) (*p<0.05 *vs.* Group D,^ +^ *p*<0.05 *vs.* Group A and B).

Bone tissue regeneration within the scaffold was evaluated by micro-CT at 12 weeks post-surgery (Fig. 5). In Group D, limited amount of bone has formed in the defects postoperatively, while in Group A, B and C, significant amount of bone formation was observed with better healing of the defect. Bony callus has formed in the interspaces between the implants and the bone tissue, and newly formed bone had tight contact with the scaffolds and infiltrated into the pore region of implants. However, in Group A and B, the ingrowth of newly formed bone tissue only limited to the peripheral region of the porous scaffolds, with little bone regeneration amount in the center of scaffold (Fig. 5A, red arrows). On the contrary, the tubular scaffolds had better bone ingrowth within the center tube, which was fully filled with new bone tissue. The ratio of bone volume/total volume (%BV/TV) in Group C (56.8±2.5%) was about 1.5-fold of Group A (37.0±3.0%) or Group B (34.8±3.0%) and 10-fold of Group D (5.9±0.9%) at 12 weeks post-surgery (n = 8 in each group, all *p*<0.05), indicating better osteogenesis of tubular scaffolds than porous or solid scaffolds (Fig. 5B, threshold of 2000 for bone tissue and 3000 for scaffold).

In addition, by comparing the micro CT images of the scaffolds before and after implantation, we observed considerable scaffold degradation occurring in vivo (Fig. 5C). At 12 weeks, the biodegradation percentages (%) of two porous scaffolds in Group A and B were 54.8±2.7% and 58.3±2.2%, both of which were remarkably faster than that of tubular scaffolds in Group C (29.0±2.8%, *p*<0.05), while Group D showed the least amount of degradation (15.9±1.8%) (Fig. 5D). The results in Fig.5 indicated that the biodegradation rate of porous scaffolds exerted a more rapid replacement than the degree of osteogenesis within the scaffolds, whereas the tubular scaffolds were resorbed at a rate corresponding to the new bone deposition.

### Biomechanical Testing

**Figure 6:**
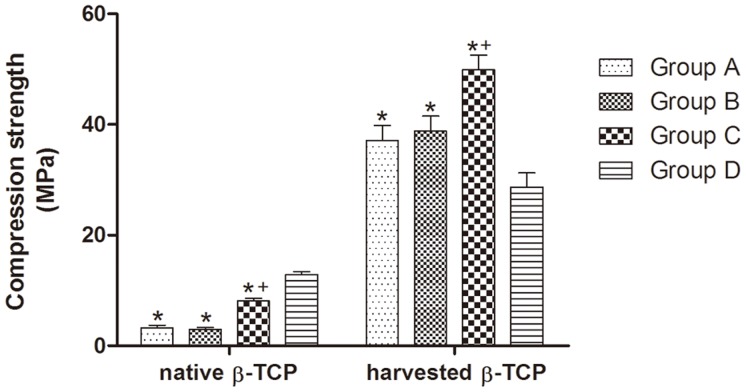
Compression testing of the scaffolds and radius defect. The results of compression testing showed that tubular scaffolds (Group C) demonstrated improved mechanical properties with higher compression strength in vivo than porous ones (Group A and B), indicating better bone formation and integration with the tubular scaffolds; the native solid scaffolds (Group D) showed the highest compression strength, but the lowest in vivo data after implantation (*p<0.05 *vs.* Group D,^ +^ *p*<0.05 *vs.* Group A and B).

In order to evaluate the mechanical properties of the scaffolds and the integration of the new bone tissue within scaffolds, we carried out compression tests on the native and implanted scaffolds 12 weeks post transplantation. The solid shaped scaffolds (Group D) showed the highest compression strength of native scaffolds but achieved the lowest strength of harvested scaffolds after 12 weeks implantation, indicating little bone formation and osteointegration with scaffolds (Fig.6). Tubular scaffolds (Group C) demonstrated significantly higher compression strength for both native (8.10±0.44 MPa) and implanted scaffolds (49.89±2.62 MPa) compared to porous ones of Group A (3.24±0.48 MPa for native and 37.12±2.13 MPa for implanted scaffolds, both *p*<0.05) or Group B (2.93±0.38 MPa for native and 38.79±2.70 MPa for implanted scaffolds, both *p*<0.05), indicating better mechanical property and improved osteointegration capability of the tubular bone grafts.

### Histological Analysis

**Figure 7:**
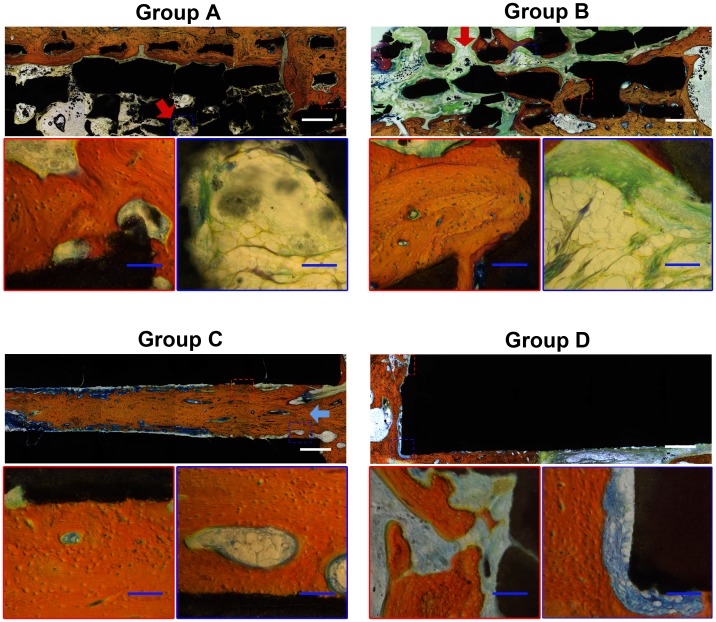
Histological analysis of new bone formation at 12 weeks post-surgery. Van Gieson staining showed more bone formed in tubular scaffolds (Group C) than porous (Group A and B) and solid (Group D) ones, especially in the center of grafts (blue arrow). The center region of porous scaffold was occupied by soft tissue with poor osteogenesis (red arrow). The tissue stained in red color was the newly formed bone with visible cell nuclei. The tissue stained in yellow/green/blue color was fibrous tissue. Scale bar: 1 mm (white), 100 µm (blue).

**Figure 8:**
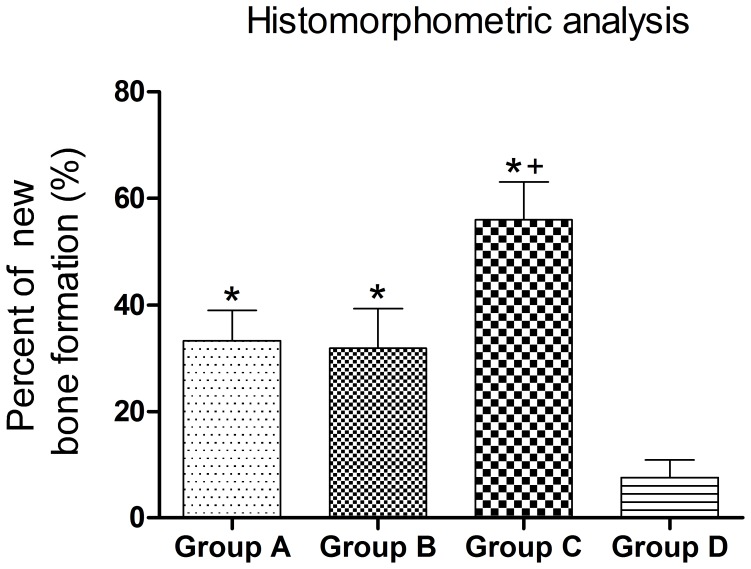
Histomorphology analysis of new bone formation. Quantitative analysis showed the percentage of new bone formation in Group C was significantly higher than that of Group A, B and D at 12 weeks post-surgery (*p<0.05 *vs.* Group D,^ +^ *p*<0.05 *vs.* Group A and B).

To evaluate the tissue response to the implanted scaffolds and the defect healing progress, we performed histological analysis on the tissue/biomaterial interface and the area of the implant. As shown in Fig. 7, there was little bone tissue in Group D, and the defects around the scaffolds were filled with fibrous tissue. The implants in Group A–C showed significantly better new bone formation, with bone trabecular structure found at 12 weeks compared to Group D. However, Group A and B experienced limited ingrowth of bone tissue, which only formed in peripheral regions of the scaffold, with the central region of the scaffold occupied by fibrous tissue (Fig. 7, red arrow). On the contrary, Group C achieved much better bone formation and bone infiltration, with central tube fully filled with newly formed bone tissue at week 12 after surgery. Furthermore, bone marrow cavity achieved recanalization in the central tube of Group C (Fig. 7, blue arrow). The percentage of new bone formation (bone area/total area) in Group C (55.9±2.9%) was significantly higher than that of Group A (33.2±2.3%), B (31.8±2.0%) and D (7.6±1.3%) (n = 8 in each group, all *p*<0.05), indicating better bone ingrowth in tubular scaffolds than other ones (Fig. 8).

### ECT Examination

**Figure 9:**
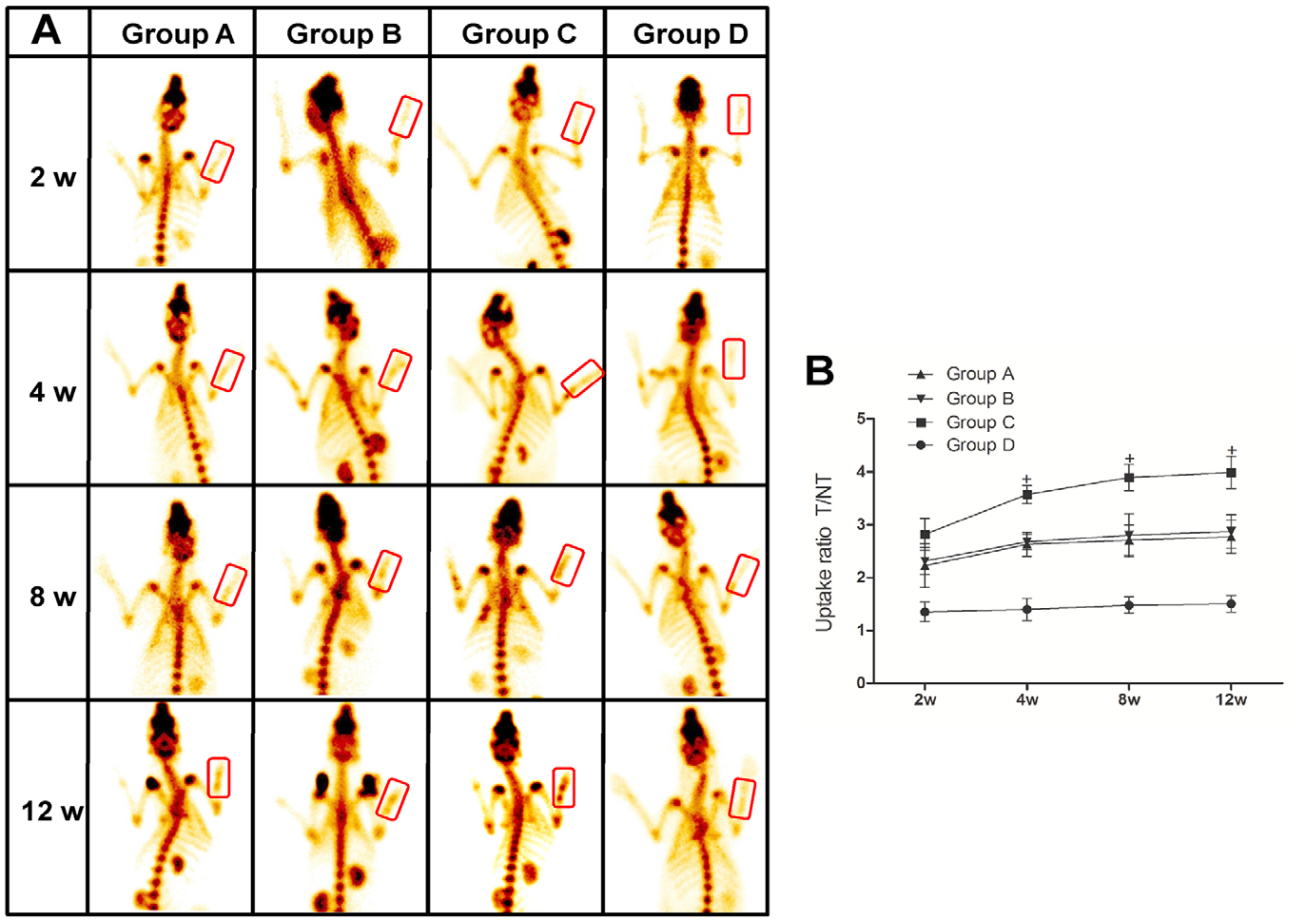
ECT analysis of vascularization at 2, 4, 8 and 12 weeks post-surgery. (**A**) ECT images of rabbits showed that despite of no difference among the four groups at 2 weeks post-surgery, higher counts were observed in tubular scaffolds (Group C) than other groups (Group A, B and D) since week 4 post-surgery, suggesting enhanced vascularization and metabolic activities. (**B**) Quantitative measurement of the uptake ratio (T/NT) further confirmed better vascularization and higher metabolic activities of tubular scaffolds than others since week 4 post-surgery (^+^ *p*<0.05 *vs.* Group A, B and D).

Bone scintigraphy with radioactive tracers has been widely used in the imaging of vascularization and metabolic activity of bone tissue. In this study, ECT was conducted at 2, 4, 8 and 12 weeks after surgery to analyze the vascularization of the TCP scaffolds. Four hours after injection of 99mTc-MDP, about half of the tracer was deposited in the bone and delayed images were obtained (Fig. 9A). The uptake ratio of ^99m^Tc-MDP (T/NT) in the defect region tended to increase with time after the surgery throughout the whole study in each group, while the rate of increase became gradually steady after 4 weeks post-surgery. The solid scaffolds (Group D) had the lowest uptake ratio which changed slightly after implantation, indicating limited infiltration of capillary vessels. Group A, B and C displayed comparable 99mTc-MDP uptake ratio (T/NT) at 2 weeks, while tubular scaffolds (Group C) had a higher degree of blood flow perfusion from 4 weeks post-surgery than that of porous scaffolds (Group A and B) (Fig. 9B). At 12 weeks post-surgery, the T/NT value of tubular scaffolds reached to peak at 4.00±0.30, which was 1.5-fold higher than that of porous ones (2.77±0.32 in Group A and 2.87±0.32 in Group B, both *p*<0.05).

## Discussion

Porous scaffolds as bone graft substitutes have been widely investigated for segmental bone defect treatment [23], [24], while their in vivo application could be harassed by the limited bone ingrowth and poor vascularization in the central region of the bone grafts when implanted in the large bone defect, especially the segmental bone defect [25], [26], [27]. The current advance in manufacturing technology facilitates the accurate control of 3D scaffold structure parameters and makes the optimization of internal structure possible [17], [28]. However, most of these studies concentrate on changing internal structural properties instead of adjusting overall architecture. In the present study, we investigated the use of tubular architecture scaffolds which mimicked the overall shape of long bone and systematically compared to porous scaffolds with different internal structures. Our results demonstrated that compared to porous scaffolds, the tubular scaffolds were more favorable for segmental bone defect treatment, demonstrating higher degree of bone tissue ingrowth and vascularization, as well as better biomechanical properties both in vitro and in vivo.

Scaffolds that mimic natural bone structures could be used as a favorable bone graft substitutes for cell and bone ingrowth when implanted, with reported efficacy in promoting in vivo osteogenesis and integration with surrounding bone tissue [29], [30], [31]. Porous scaffolds with interconnected pores have been widely investigated for bone defect treatment, since it can imitate the structure of cancellous bone and provide an open and connected porous framework for cellular migration, extracellular matrix production, new bone formation and the neo-vascularization from adjacent bone tissue [32], [33]. It is well known that the pore size is one of the most critical architecture factors, which impose profound influence on the progression of osteogenesis [14], [34], with the studies showing that 300–900 µm pore size is suitable for BTE application [35]. Porous scaffolds have been proved to be an effective bone substitute in the treatment of cavity defect which is surrounded predominantly by bone tissue [27], [36]. However, when porous scaffolds were implanted in certain defect region which is surrounded by soft tissue predominantly, such as the segmental defect, the fibrous tissue may easily outgrow the bone tissue and occupy the space for new bone formation, thus compromising the bone regeneration efficacy. In our previous study, we have observed that the implantation of porous scaffolds led to the non-union treatment of femoral segmental defect in a rat model with massive fibrous tissue invasion [25]. Zhou et al reported that only a small amount of new bone and blood vessel were formed within the porous β-TCP scaffolds, which contributed to poor biomechanical strength and failed in repairing large segmental bone defect [26]. Although 500 µm of pore size for porous scaffold was suggested in Kujala et al’s study, demonstrating a significantly less degree of fibrosis than the scaffold with smaller pore (260 µm), in our study, the implantation of 500 µm porous scaffold still led to the rapid infiltration of peri-implant fibrous tissue at early stage, which grew faster than bone tissue, as demonstrated by micro CT analysis (Fig. 5) and histology assay (Fig. 7). Besides precluding the new bone formation by occupying the defect space, the fibrous tissue influx can also generated a thick tissue layer around the surface of grafts, adversely affecting the sufficient blood perfusion, which may further jeopardize the bone regeneration process [37], [38].

When compared to porous scaffolds, tubular scaffolds demonstrated to be better bone graft substitute design in treating the segmental defect, achieving considerably better quantity and quality of new bone formation. Generally, it is more challenging to repair the segmental bone defect than a cavity bone defect, due to poor bone formation environment of segmental defect, which is surrounded predominantly by soft tissue. To prevent the rapid invasion of surrounding soft tissue and protect the defect space for subsequent bone tissue ingrowth has become the key for successful defect healing [1], [39]. Unlike porous scaffolds allowing the tissue ingrowth from all the interfaces with surrounding tissues, tubular scaffolds can selectively permit the ingrowth of surrounding tissue only from the tubular openings. When implanted into the segmental defect, the openings of tubular scaffolds are in tight contact with bone tissue; while the other interfaces contacting soft tissue is non-permeable for tissue infiltration, hence preventing the invasion of soft tissue effectively, as demonstrated by histological analysis (Fig. 7). With the well-protected space and favorable chemical surface of osteo-conductive TCP material, osteogenic cells could migrate from adjacent bone tissue, proliferate along inner wall of tubular scaffolds and deposit new bone tissue into the core region of grafts; in this way, implantation of tubular scaffolds have helped to achieve the complete union of segmental bone defect, evidenced by the X-ray examination, micro-CT analysis and histology assay (Fig. 3,5 and 7).

Moreover, tubular scaffolds facilitated higher volume of vascularization within the grafts than porous ones, as indicated by the radionuclide bone imaging. Radionuclide bone imaging is a well-established clinical examination technique to assess the abnormalities of bone tissue [26], [40], [41]. The uptake of the radiopharmaceutical depends both on an adequate delivery system and on a living network of osteocytes [42]. ^99m^Tc-MDP, the most commonly used tracers in clinical bone research, will be accumulated mainly into the bone tissue after injection, especially in the highly vascularized region and the accumulation dosage is closely correlated to the degree of vascularization. The capacity and accuracy of ECT to evaluate the vascularization has been validated by a number of other methods such as magnetic resonance imaging (MRI) [43], digital subtraction angiography (DSA) [44] and histology [26], [45]. The ECT examination showed a significantly higher uptake of 99mTc-MDP in the defect region with the implantation of tubular scaffolds throughout the whole study (Fig. 8). Although scaffolds with porous structure are thought to favor vascular ingrowth from surrounding tissue, they allow the ingrowth of fibrous tissue as well, which may adversely affect the subsequent vascularization especially in the centre space of the implants [46]. Furthermore, it has been acknowledged that the persistent peri-implant inflammation could inhibit the process of vascularization [47], [48]. As the porous scaffolds with the open structure were inevitably exposed to the surrounding environment, the sustained inflammatory reaction accompanied with traumatic condition of segmental bone defect may possibly influence the vascularization of porous scaffolds more easily than that of tubular scaffolds which provided a relatively enclosed environment to block the prolonged inflammatory reaction. This hypothesis could partially explain the finding in our study that although being similar at the first 2 weeks, the vascularization became more pronounced in tubular scaffolds than in the porous ones since 4 weeks. The histological analysis and molecular pathways examination for the specific underlying mechanism of better angiogenesis in tubular scaffolds will be conducted in our further studies.

Despite of its osteoconductive properties, the in vivo application of β-TCP is generally fraught with the problem of rapid degradation after the implantation [49], [50]. Because of its higher surface/volume ratio, β-TCP scaffold with porous structure could experience faster in vivo degradation than tubular structure, as evidenced by micro CT analysis in our study (Fig.5). This rapid degradation of porous β-TCP scaffolds could result in the early disintegration of overall structure, eliminating the space for the bone tissue formation, and the TCP degradation debris may block the porous channel for the ingrowth of vessel and bone tissues [51], [52]. On the contrary, tubular scaffolds could maintain the integrity of scaffold structure in a longer period of time for the ingrowth of the vessels and bone tissue. In addition, tubular scaffolds possessed better inherent mechanical property as shown by the in vitro compression testing (Fig.6). When implanted in vivo, it can restore significantly higher mechanical strength of the damaged bone than porous scaffolds, which could be explained by its slower degradation profile, better preservation of its structure integrity and more bone tissue ingrowth, thus demonstrating its promising potential in the load-bearing application.

### Conclusions

In this study, we have systematically compared the efficacy of tubular scaffolds with porous scaffolds for segmental bone defect treatment, and demonstrated that compared with the traditional porous structure, scaffolds with tubular structure can promote much better defect healing with significantly higher amount of bone tissue formation and neo-vascularization, greater in vivo biomechanical strength and eventually achieving the complete union of segmental defect. These findings have provided a further knowledge about the influence of scaffold architecture on their in vivo defect healing performance and illuminated the great potential to use tubular scaffolds as effective bone graft substitutes in the segmental defect treatment.
